# Au Quantum Dots Anchored Y‐MOF Enabling Directional Interfacial Charge Transfer for Synergistic Redox Photocatalysis

**DOI:** 10.1002/advs.78695

**Published:** 2026-11-05

**Authors:** Hui Yang, Dong Xu, Xue Lu, Yinbao Wu, Feng Tang, Linfu Xie, Saisai Yuan, Xiaozhi Wang, Qitao Zhang, Bin Xu

**Affiliations:** ^1^ School of Environmental Science and Engineering Yangzhou University Yangzhou China; ^2^ School of Chemistry and Materials Yangzhou University Yangzhou China; ^3^ Institute of Microscale Optoelectronics International Collaborative Laboratory of 2D Materials for Optoelectronics Science and Technology of Ministry of Education Shenzhen University Shenzhen China; ^4^ School of Environmental and Chemical Engineering Jiangsu University of Science and Technology Zhenjiang China

**Keywords:** interfacial charge migration, quantum dots, rare earth‐based MOFs, synergistic photocatalysis

## Abstract

The synergistic photocatalytic production of hydrogen coupled with pollutant degradation offers a transformative pathway toward sustainable energy and environmental remediation. Here, a novel bifunctional photocatalyst was prepared by anchoring Au quantum dots (QDs) onto a pyrene‐derived yttrium‐based metal‐organic framework (Au/YT) using a one‐pot solvothermal approach. Notably, the optimized 2.0%Au/YT demonstrated an outstanding photocatalytic redox activity for H_2_ production and toxic acetaldehyde (CH_3_CHO) removal simultaneously under visible light irradiation, surpassed 15.94‐ and 19.86‐times higher than that of original H_4_TBAPy counterpart. Comprehensive optical and electrochemical analyzes indicated that Au QDs significantly expand the light absorption range while also successfully speeding up the separation of photogenerated carriers. Meanwhile, the intrinsic multilayered structure of Y‐MOFs further synergizes with the anchored Au‐QD sites to establish metal–support interactions and augment charge transport dynamics, leading to a remarkable improvement in collaborative photocatalytic efficiency. Additionally, in situ XPS tests and DFT calculations substantiated a robust electronic interplay at the Au/YT interface, which effectually orchestrates directed interfacial charge migration and optimizes the adsorption/activation energy of reactant molecules. This work pioneers the development of dual‐functional rare earth‐based MOFs photocatalysts and furnishes fundamental insights into the rational design of synergistic redox photocatalysis systems tailored for clean solar energy production and environmental stewardship.

## Graphical abstract

Metal‑support interaction and directional interfacial charge transfer in Au QDs‐anchored Y‐MOF synergistically greatly boost redox photocatalysis for hydrogen evolution and acetaldehyde removal.

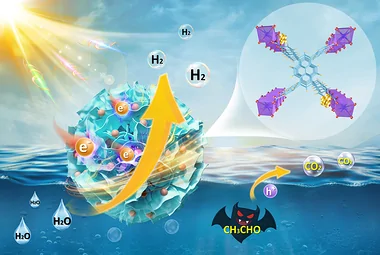

## 1 Introduction

The escalating global energy crisis, compounded by relentless environmental deterioration, constitutes one of the most formidable challenges confronting contemporary society [1, 2]. In response, most of scientific research fields are pivoting toward sustainable and green technologies that hold the promise of concurrently alleviating energy shortages and mitigating pollution [3, 4]. Among various proposed solutions, photocatalysis has emerged as a promising approach as it enables the direct transduction of abundant solar energy into storable chemical energy under mild conditions, while avoiding secondary contamination [5, 6, 7]. As a matter of priority, current frontiers are oriented toward amplifying collaborative photocatalytic efficiency to achieve full utilization of photogenerated carriers (electrons and holes), exemplified by the reductive generation of hydrogen (H_2_) from water splitting and the oxidative degradation of environmental pollutants [8, 9].

In the pursuit of high‐efficiency photocatalysts, metal‐organic frameworks (MOFs) have garnered escalating interest in recent years [10, 11, 12]. Constructed structure via the self‐assembly of inorganic nodes and organic struts, MOFs are distinguished by exceptionally high specific surface areas, structural diversity, and tailorable porosity. These features enable robust reactant adsorption, furnish abundant catalytic centers/sites, and permit precise modulation of optoelectronic properties for diverse applications [13, 14]. Within this family, rare‐earth‐based MOF (RE‐MOF) materials are prominent for their unique photophysical properties and superior chemical/thermal robustness [15, 16]. For the majority of RE ions with partially filled 4f orbitals, unpaired 4f electrons contribute to multivalent and multi‐coordination complexes [16, 17]. Recent advances in RE‐MOFs have provided versatile platforms through coordinative, covalent, and supramolecular interactions [18, 19, 20, 21]. For example, Wang et al. constructed a mixed polynuclear cluster‐based Y‐MOF with a novel (12, 12, 18)‐connected topology for efficient propylene/ethylene separation and iodine adsorption [22]. Unlike most RE ions, Y^3+^ has a [Kr] 4d^0^4f^0^ configuration with empty 4d/4f orbitals; thus, photocatalysis in Y‐MOFs is generally governed by ligand‐to‐metal charge transfer (LMCT) and π‐conjugated ligand absorption, rather than by unpaired 4f electrons [23, 24]. Although Y‐MOFs show structural versatility, studies have mainly focused on separation/adsorption of environmental pollutions, leaving photocatalysis largely underexplored and motivating the present work [23, 24].

Notwithstanding these inherent merits, the photocatalytic output of pristine MOF remains severely circumscribed by two intrinsic bottlenecks: a restricted light absorption ability that underutilizes the solar spectrum, and a pronounced propensity for photogenerated electron–hole pair recombination, which curtails the abundance of available surface‐active charge carriers [25, 26, 27]. When metal ions are combined with organic linkers of π‐conjugated structure, the designed MOF manifests augmented light‐harvesting capacity and expeditious charge transport, positioning it as an ideal candidate for photocatalysis [23, 24]. Prior investigations have corroborated that introducing noble metals into the MOF interface can establish a Schottky barrier, thereby markedly suppressing charge recombination [28, 29, 30]. In addition, some researches have shown that the impregnation of noble metal species into the MOF matrix can reshape the electronic band architecture and amplify the photo‐redox potency through strategic shifting of band‐edge potentials [31, 32]. For example, Baluk et al. demonstrated that Cu and Ag‐metal‐modified MOFs can broaden the photo‐response range and accelerate electron transfer between the metal‐MOF interface, thus enhancing the photoreduction performance of carbon dioxide conversion to formic acid [33]. Such a strategy facilitates an in‐depth exploration of the synergistic coupling between the MOF host and noble metal guests, thereby furnishing a theoretical cornerstone for the rational refinement of photocatalytic systems [34, 35].

In this work, by using a one‐pot solvothermal technique to attach Au quantum dots (QDs) into a yttrium‐based MOF (referred to as Au/YT), a novel bifunctional photocatalyst was fabricated. The central objectives are focused in this study. One target is to systematically unravel how Au QDs modulate the electronic landscape and charge‐transfer kinetics of the parent Y‐MOFs, and the other is to critically evaluate the performance of Au/YT in the concurrent photoreduction of water to H_2_ and photooxidation of toxic acetaldehyde (CH_3_CHO) to CO_2_. As is known to all, CH_3_CHO has been classified as a Group I carcinogen by the International Agency for Research on Cancer (IARC). By synergizing comprehensive experimental characterizations with theoretical density functional theory (DFT) computations, the interfacial charge‐transfer trajectory was elucidated and a cogent structure‐activity correlation was established. This work not only propels the development of high‐performance, bifunctional MOFs‐based photocatalysts but also yields pivotal mechanistic insights into charge‐carrier behavior within noble‐metal‐anchored RE‐MOFs materials, providing invaluable guidance for the rational design of advanced photocatalysts for energy conversion and environmental remediation applications.

## 2 Experimental Section

### 2.1 Synthesis of Au QDs‐Anchored Y‐MOF (Au/YT) Photocatalyst

In a representative synthesis process, 136.53 mg of 1, 3, 6, 8‐tetra(4‐carboxyl phenyl) pyrene (H_4_TBAPy, 0.2 mmol), 114.87 mg yttrium nitrate (Y(NO_3_)_3_, 0.3 mmol) and 6.0 µmol gold(III) chloride hydrate (HAuCl_4_) were co‐dissolved in 20.0 mL of a mixed solvent (1, 4‐dioxane/DMF/H_2_O with a volumetric ratio of 1:3:1). After being magnetically agitated at 25°C for 3 h, the mixture was placed in a 50 mL Teflon‐lined stainless‐steel autoclave and treated with a solvothermal treatment at 120°C for three days. Upon cooling, the precipitate was collected by centrifugation, washed repeatedly with a DMF/H_2_O mixture (1:1 by volume) at 10000 rpm for 15 min per cycle (at least three times), and finally dried at 80°C for 12 h to afford a yellow powder of gold quantum dots (Au QDs)‐anchored Y‐TBAPy (denoted as Au/YT). Typically, about 0.14 g of the final product was obtained, corresponding to a yield of ca. 85% with respect to both the yttrium precursor and H_4_TBAPy ligand. Furthermore, to systematically vary the Au QD loading, different volumes of HAuCl_4_ solution were introduced to yield nominal molar ratios of 0, 0.5%, 1.0%, 2.0% and 3.0% (denoted as xAu/YT). Prior to photocatalytic testing, each sample was subjected to UV irradiation for 12 h to eliminate any surface‐adsorbed organic residues.

### 2.2 Collaborative Photocatalytic Redox Test of H_2_ Evolution and CH_3_CHO Decomposition

Synergistic photocatalytic redox reactions were conducted in an automated online trace gas circulation system (Beijing Perfectlight, Labsolar‐6A) associated with a gas‐tight glass reactor. Typically, 20 mg of photocatalyst was dispersed into 40 mL H_2_O solution containing 0.4 mL of acetaldehyde (CH_3_CHO), without an additional sacrificial agent. The illumination source used a 300 W Xe lamp (Beijing Perfectlight, PLS‐SXE300D) with a 420‐nm cutoff filter. The reaction temperature was rigorously maintained below 15°C via a continuous condensate recirculation system. Maintaining the reaction temperature below 15°C minimizes CH_3_CHO volatilization, ensuring a reliable liquid‐phase CH_3_CHO concentration. Finally, the evolved H_2_ (generated from water splitting) and liberated CO_2_ (originating from CH_3_CHO mineralization) were quantitatively analyzed in real time using the matched gas chromatography (FULI, GC979 Plus, equipped with TCD detectors).

## 3 Results and Discussion

### 3.1 Structural and Morphological Characterizations

X‐ray diffraction (XRD) was typically used to assess the crystallographic phase and purity of the as‐prepared samples. As seen in Figure 1a, all samples had distinct diffraction peaks, demonstrating their great phase purity and crystallinity [36]. For the xAu/YT series photocatalysts, characteristic peaks corresponding to both metallic Au substance and the pristine Y‐MOF were simultaneously observed. Specifically, these evident peaks situated at 38.23°, 44.42°, 64.65°, and 77.59° were assigned to the (111), (200), (220), and (311) crystallographic planes of face‐centered cubic Au (JCPDS No. 04–0784), respectively [32, 37]. Meanwhile, the intensity of these Au characteristic peaks is proportional to the amount of Au precursor added, confirming the progressive incorporation of Au species. ICP‐OES measurements were performed to determine the actual Au loadings; the tested Au contents were 0.34, 0.71, 1.43, and 2.11 wt.% for 0.5%Au/YT, 1.0%Au/YT, 2.0%Au/YT, and 3.0%Au/YT, respectively (Table S1). Previous studies have indicated that each yttrium (Y) atom coordinates with eight carboxylate oxygen (O) atoms from four H_4_TBAPy ligands to form a robust secondary building Y‐MOF unit (Figure S1) [23, 36]. These units assemble into layered structures, and these multiple layers will systematically arrange into a 3D extended network [30, 31]. Apparently, the introduction of Au quantum dots (QDs) is expected to facilitate rapid electron shuttling between the stacked layers, substantially improving charge separation efficiency on the Y‐MOF photocatalyst surface [22, 37].

**FIGURE 1:**
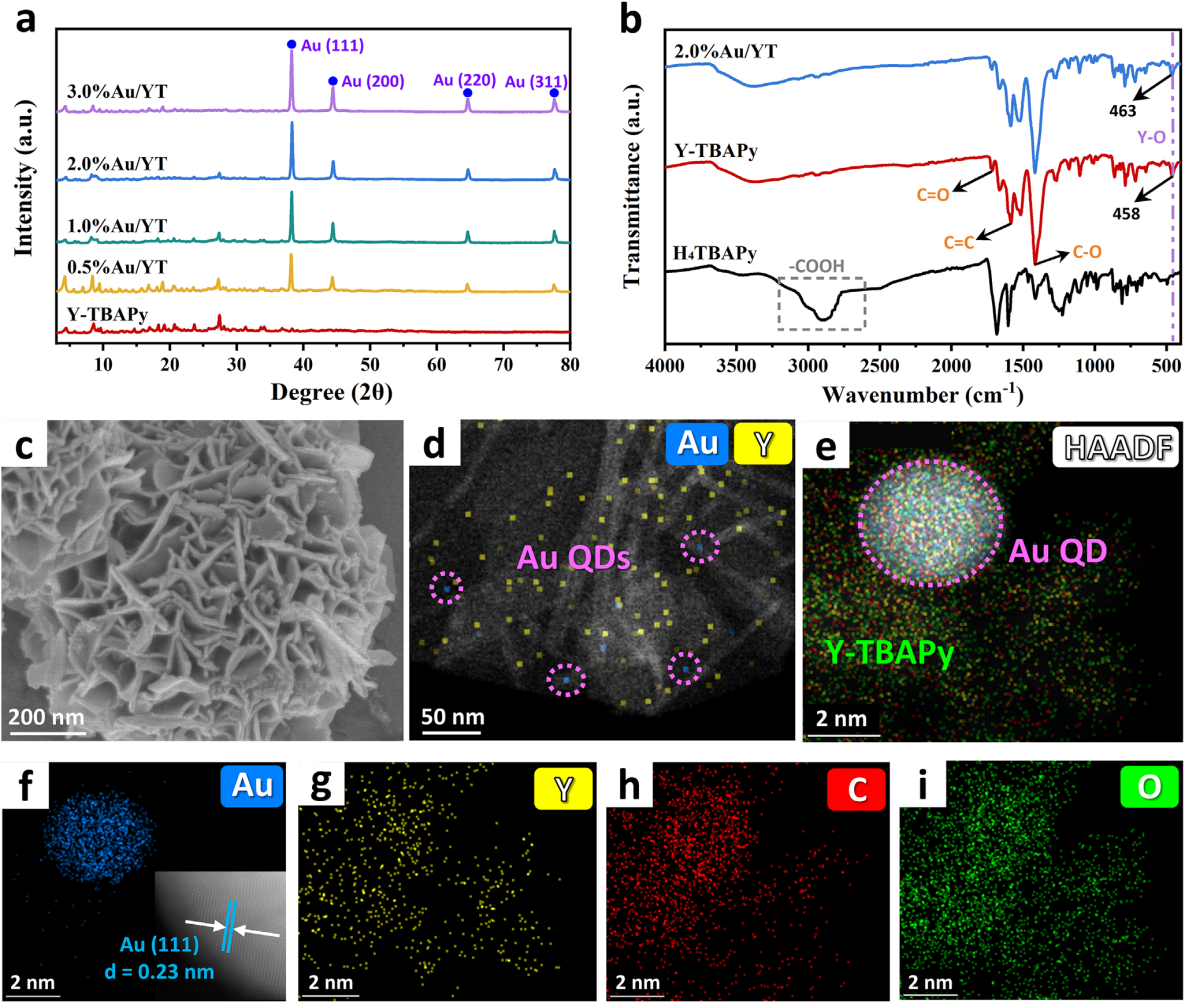
(a) XRD pattern of YT‐based photocatalysts; (b) FT‐IR spectra of 2.0%Au/YT, Y‐TBAPy and H_4_TBAPy, respectively; (c) SEM image; (e) HAADF image and (d, f‐i) Elements distribution mapping images of 2.0%Au/YT photocatalyst.

The chemical bonding environment of the photocatalysts was assessed using Fourier transform infrared spectroscopy (FT‐IR) technology (Figure 1b). The hydroxyl vibration of the –COOH group was identified as the cause of a notable peak in the H_4_TBAPy ligand in the 2700–3100 cm^−1^ range [17, 18]. This peak vanished in the Y‐TBAPy and 2.0%Au/YT, indicating that the hydroxyl broke down during the synthesis process. In the meantime, relative to the original H_4_TBAPy ligand, a new vibration peak appeared at 458 cm^−1^ for pristine Y‐TBAPy, evidently confirming the successful formation of Y─O coordination bonds [36]. In the case of the 2.0%Au/YT sample, the vibrational band displayed a noticeable shift to 463 cm^−1^, indicating a strong electronic perturbation and chemical interaction between Au QDs and Y‐MOF components. This phenomenon also signified the formation of chemical bonds cooperation rather than simple physical adsorption in the 2.0%Au/YT photocatalyst. Moreover, the vibrational peaks located at 1417, 1585, and 1713 cm^−1^ in both 2.0%Au/YT and Y‐TBAPy were attributed to the presence of intact carboxylic acid groups in Y‐MOF [36]. Notably, the vibration peak at 1589 cm^−1^ in 2.0%Au/YT showed a blue shift compared to that of pure Y‐TBAPy (1585 cm^−1^), this shift fully validated the successful construction of the ‐COO‐Y coordination structure and the effective electronic integration of Au QDs within the Y‐MOF photocatalyst.

The morphology and elemental distribution of the produced materials were investigated using scanning electron microscopy (SEM) and high‐resolution transmission electron microscopy (HR‐TEM) (Figure S2). As depicted in Figure 1c, the Au quantum dot‐anchored yttrium‐based metal‐organic framework (Au/YT) sample displayed a complex multilayer flower‐like morphology, with a diameter of about 1–2 µm, consisting of interleaved petal‐like nanosheets. This unique hierarchical morphology is anticipated to provide a high surface area and facilitate mass transport, both of which are beneficial for photocatalytic reactions. Energy‐dispersive X‐ray (EDX) elemental mapping images revealed the presence of Au, Y, C, and O elements across the entire architecture of the Au/YT photocatalyst. Interestingly, the element of Au appeared as localized bright spots measuring about 2–3 nm in size, well indicating the quantum dot (QD) configuration (Figure 1d‐e). Furthermore, the uniform co‐localization of Au with the other elements confirmed the structural integrity of the MOF framework after Au QDs incorporation (Figure 1f‐i). In summary, the one‐pot solvothermal method employed in this work effectively enabled the successful fabrication of an Au QDs‐anchored yttrium‐based MOF photocatalyst. The Au QDs are also uniformly dispersed across the entire architecture and co‐localize perfectly with the other elements, further corroborating the successful and homogeneous anchoring of Au QDs onto the Y‐MOF support.

### 3.2 Exploration of Surface Chemical States and Electron Transfer Behavior

The surface chemical states and interfacial charge‐transfer behavior were investigated using X‐ray photoelectron spectroscopy (XPS) measurements (Figure S3). Analysis of survey spectra clarified that the pristine Y‐TBAPy photocatalyst comprised of Y, O, and C elements, while the introduction of Au QDs resulted in a distinct Au signal in 2.0%Au/YT (Figure 2a). High‐resolution C 1s spectra can be deconvoluted into four components at binding energy (B.E.) of 290.16 eV (‐COO), 288.51 eV (C═O), 286.11 eV (O‐C‐Y), and 284.49 eV (C‐C), respectively (Figure 2b) [36, 38]. The successful coordination effect between yttrium ions (Y^3+^) and H_4_TBAPy ligands is confirmed by the appearance of the C‐O‐Y signal. More importantly, upon Au QDs loading, the C═O and C‐O‐Y peaks display a perceptible negative shift in binding energy, implying that the electronic perturbation primarily localizes on the carboxylate carbons rather than the aromatic ring carbons (C‐C). For the separated spectra of O 1s on 2.0%Au/YT sample, peaks located at B.E. of 532.87, 531.60, and 530.57 eV were assigned to C‐O, C═O, and Y‐O/Au‐O, respectively (Figure 2c) [39]. Upon Au QDs loading, the systematic shift of O 1s and C 1s peaks to lower binding energy pointed to the strong electronic coupling and charge redistribution at the Au/YT interface, also confirming the intimate interactions between Au QDs and pristine Y‐MOF components. Furthermore, the peaks situated at B.E. of 158.29 eV (Y 3d_3/2_) and 156.21 eV (Y 3d_5/2_) were observed in the Y 3d spectra for 2.0%Au/YT, while the positive shifts observed for Au/YT sample provided strong evidence of the robust interaction between Au QDs and YT components again (Figure 2d) [22, 38]. In addition, the Au 4f spectra exhibited two prominent peaks at B.E. of 87.60 eV (Au 4f_5/2_) and 83.92 eV (Au 4f_7/2_), evidently affirming the predominant presence of metallic gold (Au^0^) (Figure 2e) [32, 37]. The successful attachment of Au QDs signified that strong interactions between the two‐phase interface could optimize the dynamics of photogenerated carriers, ultimately enhancing the photoredox efficiency compared to Y‐TBAPy.

**FIGURE 2:**
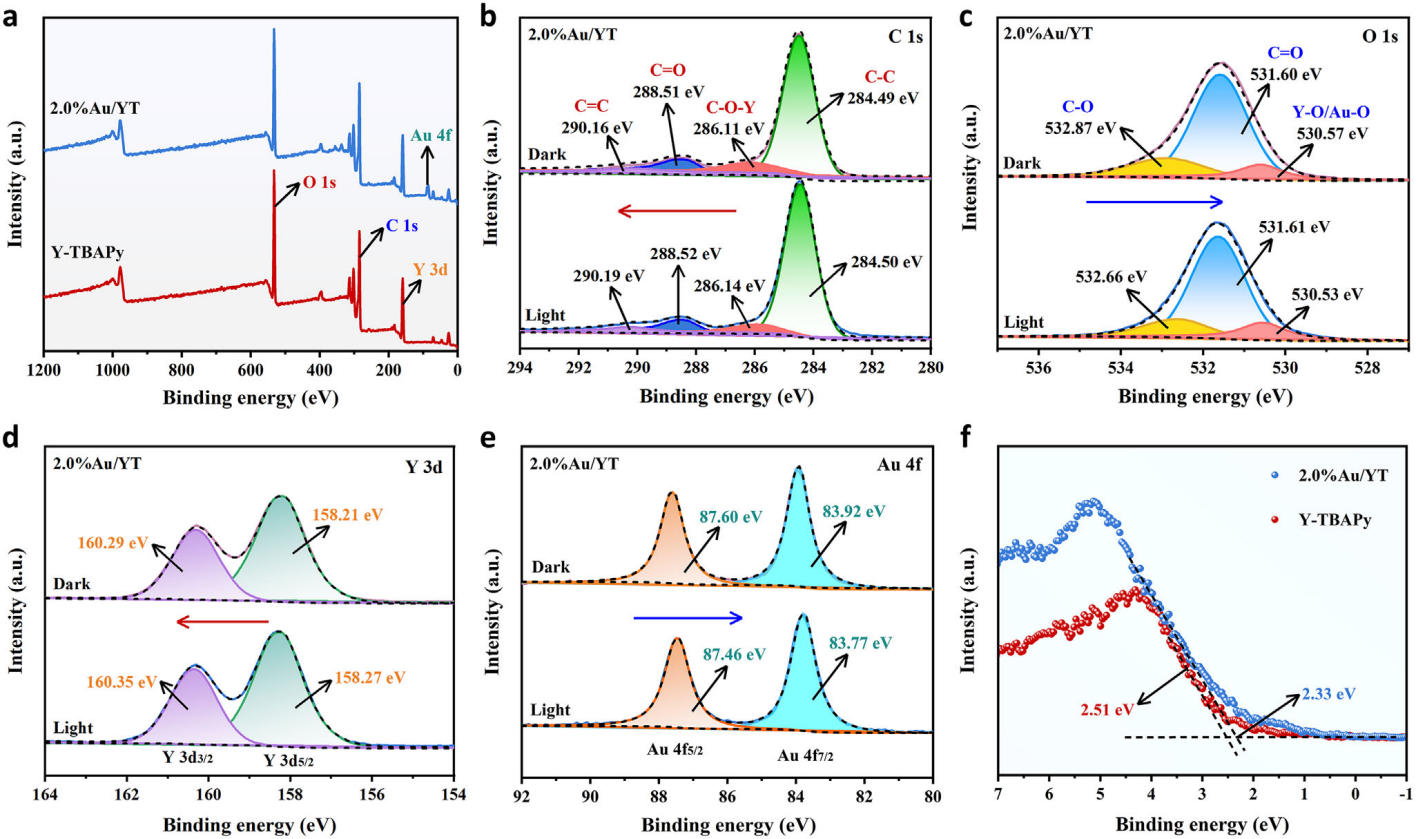
(a) Wide scanning XPS survey spectra of Y‐TBAPy and 2.0%Au/YT; In situ XPS spectra and high‐resolution deconvoluted curves (b) C 1s; (c) O 1s; (d) Y 3d; (e) Au 4f of 2.0%Au/YT photocatalyst and (f) VB‐XPS spectra of 2.0%Au/YT and pristine Y‐TBAPy.

In situ XPS measurements were carried out in both dark and visible light illumination settings to provide a deeper understanding of the photoinduced charge transfer pathway between the Au QDs and Y‐MOF components [36, 39]. As seen in Figure 2b, when illuminated by light, the C 1s peaks of 2.0%Au/YT exhibit an obvious shift toward higher B.E., indicating a decrease in electron density around the carboxylate‌ carbon atoms. Meanwhile, the Y 3d peaks also shift to higher B.E., implying that the yttrium sites lose electrons and become electron‐depleted (Figure 2d). In contrast, all O 1s and Au 4f display a clear shift toward lower B.E. after light illumination, implying demonstrating that the oxygen species and Au QDs gain electrons and become electron‐rich sites (Figure 2c,e). This observation, when combined with the direction of shifts seen in the C 1s and Y 3d spectra, strongly suggests a directional electron transfer from the Y‐MOF framework (particularly from the Y–O clusters) toward the anchored Au QDs side. Collectively, the opposite shift directions of lower B.E. for O and Au versus higher B.E. for C and Y reveal that photogenerated electrons are transferred from the yttrium sites to both the carboxylate carbon/oxygen atoms and the anchored Au QDs. The accumulated electrons on Au sites can subsequently participate in surface photocatalytic reduction reactions, thereby significantly boosting the photoredox performance.

### 3.3 Investigation of Optical Absorption, Photoelectrochemical Properties and Carrier Dynamics

The optical absorption characteristics were evaluated by ultraviolet‐visible (UV–vis) reflectance spectroscopy (Figure 3a). Pristine Y‐TBAPy displays a steep absorption edge at around 485 nm, characteristic of the intrinsic π–π* transition of the pyrene backbone. Meanwhile, after Au QDs incorporation, all xAu/YT photocatalysts exhibited notably higher visible light absorption than that of pristine Y‐TBAPy, especially in the region from 500 to 600 nm. This observation demonstrates that the introduction of Au QDs can effectively broaden the spectral sensitivity and enhance visible light absorption and utilization efficiency [33, 40]. Furthermore, the absorption edge of the xAu/YT also showed a slight red‐shift compared to pristine Y‐MOF, suggesting electronic interaction between Au QDs and Y‐TBAPy components that subtly alters the band structure. The band gaps of 2.0%Au/YT and pristine Y‐TBAPy were derived from the Kubelka–Munk‐transformed diffuse reflectance spectra, yielding 2.71 and 2.77 eV, respectively (Figure S4). These results indicate that anchoring Au QDs can narrow the band gap of the original Y‐TBAPy, with the band gap energy (E_g_) serving as a critical parameter for evaluating light absorption and solar energy conversion efficiency. Meanwhile, the Mott–Schottky analysis of 2.0%Au/YT exhibited a negative slope, confirming its p‐type semiconducting behavior (Figure S5). For a p‐type semiconductor, the valence‐band (VB) edge was estimated from the flat‐band potential with a typical offset. The resulting band‐edge alignment (VB = +2.33 eV, CB = −0.38 eV vs RHE), further corroborated by the VB‐XPS spectra and UV–vis analyses, is thermodynamically favorable for both proton reduction and CH_3_CHO oxidation (Figure 2f).

**FIGURE 3:**
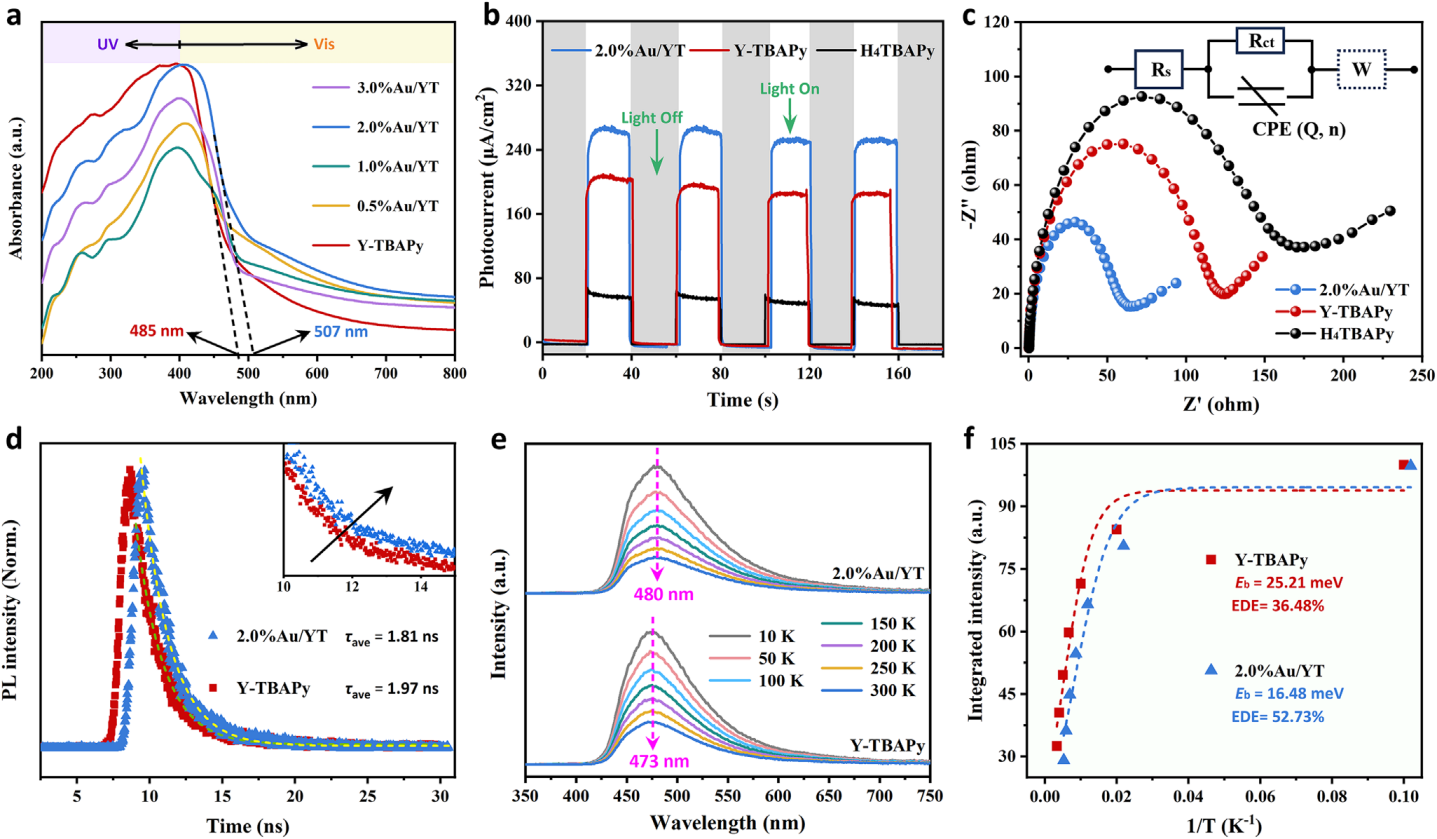
(a) UV–vis spectra of YT‐based photocatalysts; (b) Transient photocurrent response; (c) EIS Nyquist plots (inset: equivalent circuit); (d) TRPL spectra; (e) TD‐PL spectra; and (f) Integrated PL emission intensity as a function of temperature from 10 to 300 K of 2.0%Au/YT and pristine Y‐TBAPy.

The photoexcited charge separation efficacy and transfer efficiency at the photocatalyst interface were probed by transient photocurrent response measurements. As illustrated in Figure 3b, the photocurrent density of 2.0%Au/YT was recorded at 267 µA/cm^2^, representing 4.5‐times increase compared to original H_4_TBAPy (59 µA/cm^2^). This heightened tendency of photocurrent intensity suggests that 2.0%Au/YT exhibits enhanced photocarrier separation and transport capability within the bulk [41, 42]. As a consequence, this notable improvement of photocurrent density directly contributes to the enhanced synergistic photocatalytic ability of 2.0%Au/YT. Additionally, charge transfer resistance at the electrode/electrolyte interface was further investigated using electrochemical impedance spectroscopy (EIS) analysis. The equivalent circuit of R_s_+(R_ct_∥CPE), used for fitting the EIS data, is shown in the inset of Figure 3c. For 2.0%Au/YT and Y‐TBAPy, a Warburg diffusion element (W) was additionally included to fit the low‐frequency tail. Meanwhile, the corresponding fitted parameters are listed in Table S2. In the EIS Nyquist plots, the charge‐transfer resistance (R_ct_) is represented by the semicircle diameter. The smaller semicircle observed for 2.0%Au/YT compared with those for pristine Y‐TBAPy and H_4_TBAPy indicates a significantly lower R_ct_, demonstrating that the incorporation of Au QDs facilitates faster interfacial charge‐transfer kinetics and enhances electron diffusion mobility [41, 42]. In conclusion, these photoelectrochemical findings supported the synergistic effect of Au QDs and Y‐MOF in increasing charge separation, light absorption, and interfacial transfer efficiency, which in turn improved the effectiveness of synergistic photocatalysis.

Moreover, the efficiency of photogenerated charge carrier separation and transfer is a key determinant of photocatalytic performance. As shown in steady‐state photoluminescence (PL) results, pristine Y‐MOF exhibited a strong emission peak centered at around 473 nm, with a shoulder at 501 nm (Figure S6). This emission originates from the radiative recombination of photogenerated electron‐hole pairs. After Au QDs anchoring, the PL intensity of 2.0%Au/YT decreased dramatically, indicating that Au QDs can provide an alternative and efficient pathway for charge separation, effectively suppressing their radiative recombination [43, 44]. Strikingly, in comparison with pristine Y‐TBAPy, the slight red shifts to 480 and 508 nm in the 2.0%Au/YT further validate the reliability of the visible‐light response enhancement observed in UV–vis spectra analysis. To quantitatively unravel the photogenerated carrier separation and recombination kinetics, time‐resolved photoluminescence (TRPL) decay measurements were also performed. As depicted in Figure 3d, the TRPL decay of 2.0%Au/YT shows noticeably accelerated kinetics with an average lifetime of 1.81 ns, which is shorter than that of pristine Y‐TBAPy (1.97 ns). This diminished TRPL lifetime observed in the 2.0%Au/YT sample implies the operative quenching of the radiative recombination pathway, which is rationally ascribed to the rapid electron injection from the photoexcited Y‐MOF into the Au QDs [35, 36]. Consistent with the optical spectral analysis, the TRPL outcomes demonstrate that the 2.0%Au/YT photocatalyst facilitates superior separation of photoinduced carriers, consequently augmenting its efficiency in synergistic photocatalysis. The shorter lifetime in the 2.0%Au/YT suggests the emergence of a new, faster decay pathway for the photoexcited electrons, in which electrons mainly transfer from Y‐MOF to Au QD sites. This rapid interfacial electron injection is a direct manifestation of the efficient charge separation process.

Temperature‐dependent photo‐luminescence (TD‐PL) spectra were examined in order to gain a deep understanding of how the metal‑support interaction (MSI) encourages exciton dissociation (Figure 3e). The dissociation of excitons is accelerated by the temperature rise, which lowers the intensity of photoluminescence. The formula “I(T) = I_0_/(1 + Ae^−Eb/(kb^ *^T^* ^)^)” was used to obtain the exciton binding energy (E_b_), where I_0_ is the PL intensity at 0 K, k_b_ is the Boltzmann constant, and k_b_ *_T_* = 25 meV at ambient temperature [44, 45]. The exciton dissociation efficiency (EDE) of Y‐TBAPy was determined to be 36.48% (EDE = e^−Eb/(kb^ *^T^* ^)^), with an E_b_ of 25.21 meV. In contrast, 2.0%Au/YT exhibited a significantly lower E_b_ of 16.48 meV, indicating that the addition of Au QDs enables catalytic reactions at room temperature (<25 meV). Meanwhile, the much higher EDE value of 52.73% for 2.0%Au/YT suggests that interfacial charge migration between Au QDs and Y‐TBAPy components significantly enhances exciton dissociation into free carriers (Figure 3f). Moreover, this phenomenon indicates that the transfer and separation efficiency of these free electrons and holes to the 2.0%Au/YT photocatalyst surface were accelerated by the MSI. As a result, much more photogenerated free carriers in the 2.0%Au/YT photocatalyst will be involved in the subsequent photocatalytic redox reactions, ultimately leading to an enhanced photocatalytic performance.

### 3.4 Measurement of Collaborative Photocatalytic Redox Performances

Without the use of Pt co‐catalyst, the photoreduction activity was first evaluated for the evolution of hydrogen (H_2_) from water. As presented in Figure 4a, the H_2_ generation from water exhibited a near‐linear increase with irradiation time. Under visible light (Xe lamp, λ>420 nm) exposure for 2 h, 2.0%Au/YT demonstrated a significantly higher H_2_ production amount of 1017.1 µmol·g^−1^ compared to pristine H_4_TBAPy, which yielded only 63.8 µmol·g^−1^. After the normalized calculation, 2.0%Au/YT achieved an optimal H_2_ production rate of 508.5 µmol·g^−1^·h^−1^, which represented about 15.94‐times and 3.64‐times enhancement over that of pristine H_4_TBAPy and Y‐TBAPy counterparts (Figure 4b). The activity profile followed a volcano‐type trend with Au QDs loading, where 2.0% was optimal. Lower loadings (0.5%, 1.0%) provided insufficient active sites or electron sinks, while excessive loading (3.0%) may shield the Y‐TBAPy surface from light or introduce recombination centers. The wavelength‐dependent apparent quantum yield (AQY) of 2.0%Au/YT at 365, 400, 420, 500 and 550 nm was 7.36, 7.82, 7.25, 6.07 and 4.81%, respectively (Figure 4c). The AQY trend matches the UV–vis absorption spectra of 2.0%Au/YT, confirming its light‐response range is consistent with the photocatalytic activity. Under comparable conditions, the 2.0%Au/YT photocatalyst exhibits an outstanding H_2_ evolution rate and pollutant removal efficiency, demonstrating performance competitive with that of representative reported photocatalysts (Tables S3 and S4).

**FIGURE 4:**
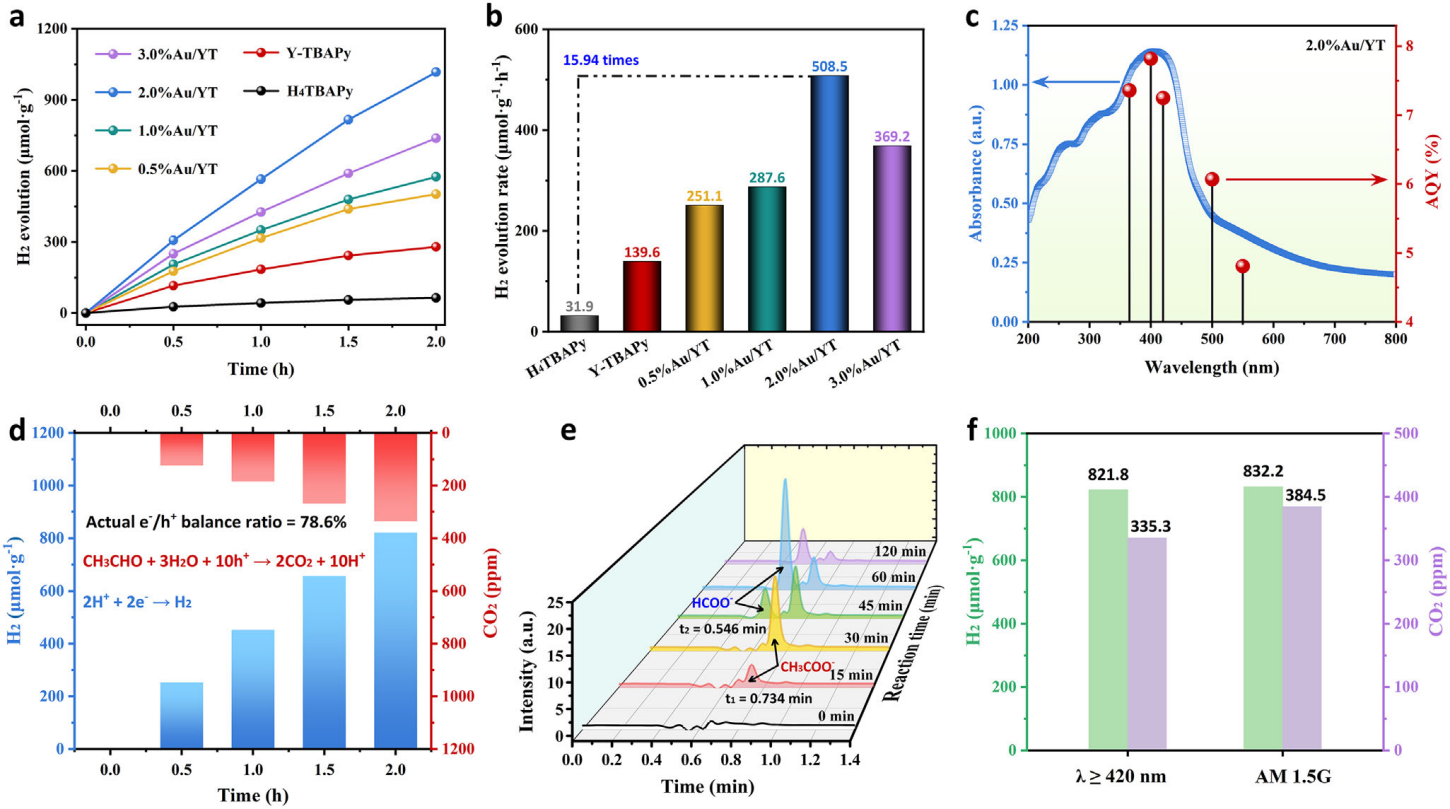
(a) Time course of H_2_ liberation; (b) H_2_ evolution rate; (c) Wavelength‐dependent AQY values of 2.0%Au/YT; (d) Collaborative photoredox performance on 2.0%Au/YT photocatalyst for H_2_O photoreduction along with CH_3_CHO photooxidation removal; (e) HPLC analysis for the collaborative photocatalytic reaction solution; and (f) Synergistic redox photocatalysis efficiency of 2.0%Au/YT photocatalyst under comparable light conditions. (Reaction conditions for a‐c: 20 mg catalyst + 40 mL H_2_O solution containing 4 mL TEOA as sacrificial agent; d‐f: 20 mg catalyst + 40 mL H_2_O solution containing 0.4 mL CH_3_CHO, without additional sacrificial agent).

To further verify the superiority of Au QDs, the performance with that of Pt nanoparticles loaded onto Y‐TBAPy (Pt@Y‐TBAPy) and Au/YT (Pt@2.0%Au/YT) was further compared under identical conditions (Figure S7). Interestingly, while Pt is generally regarded as a benchmark co‐catalyst for hydrogen evolution, the Pt@Y‐TBAPy sample exhibited a notably lower H_2_ production rate of only 377.3 µmol·g^−^^1^·h^−^^1^ under the same visible‐light irradiation. This contrast underscores that the interfacial charge transfer effect of Au QDs is more crucial to the migration and separation of photogenerated charge carriers in the 2.0%Au/YT system. In addition, the stability and recyclability of the optimal 2.0%Au/YT photocatalyst were assessed over four consecutive cycling experiments, as depicted in Figure S8. The 2.0%Au/YT photocatalyst maintained a consistently stable H_2_ evolution activity, with no significant loss of activity, demonstrating excellent photostability and reusability. The structural integrity of the spent catalyst was further confirmed by XRD analysis (Figure S9). The diffraction peaks of the 2.0%Au/YT sample after four consecutive photocatalytic cycles matched perfectly with those of the fresh sample, with no detectable shifts, phase transitions, or emerging impurity peaks. This result definitively corroborates that the crystalline framework of Y‐MOF and the anchored Au QDs remain highly robust against photo‐corrosion throughout the prolonged reaction process. Also, after the photocatalytic cycling tests, the Au 4f_5/2_ and 4f_7/2_ peaks remain at 87.62 and 83.93 eV, respectively, which are characteristic of metallic Au^0^. No significant peak shift or new Au species are observed compared with the fresh sample, indicating that the Au QDs remain metallic and stable under illumination and photoredox conditions (Figure S10).

More importantly, except for H_2_ production, the dual‐functional nature of the 2.0%Au/YT photocatalyst was also tested by measuring its ability to decompose gaseous acetaldehyde (CH_3_CHO), a typical model pollutant. As shown in Figure 4d, the CO_2_ concentration means a complete mineralization and rises steadily, the value reaching about 335.3 ppm after 2 h of reaction. This near‐stoichiometric conversion of CH_3_CHO to CO_2_ confirms efficient mineralization of the toxic pollutant. Concurrently, the system maintained a substantial H_2_ production amount of approximately 821.9 µmol·g^−^^1^ during this CH_3_CHO photooxidation process. Although this rate is slightly lower than that achieved in pure water splitting (1017.1 µmol·g^−^^1^) due to the competitive consumption of photogenerated holes by CH_3_CHO, the substantial H_2_ yield confirms that the photogenerated electrons are effectively preserved for proton reduction. This simultaneous utilization of electrons (for H_2_ production) and holes (for CH_3_CHO mineralization) firmly validates the exceptional dual‐functional photoredox capability of the 2.0%Au/YT system. The reaction intermediate during the dual‐functional reaction process was investigated by high‐performance liquid chromatography (HPLC) analysis (Figure 4e). After collaborative photocatalytic redox reactions for 2 h, this reaction system has distinct signals of both formic acid and acetic acid. Also, 2.0%Au/YT has an obvious decomposition effect on formic acid and acetic acid as time goes on, indicating that they are intermediate products capable of further oxidation and mineralization. More importantly, this conclusion is also consistent with the results of the subsequent in situ DRIFTS analysis. Moreover, after 2 h of irradiation under AM 1.5G simulated solar light, the H_2_ evolution yield still reached 832.2 µmol·g^−1^, and the CO_2_ concentration generated from CH_3_CHO mineralization reached 384.5 ppm (Figure 4f). These results support the potential applicability of Au/YT under solar‐light conditions.

Additionally, the photocatalytic oxidation efficiency of eliminating gaseous CH_3_CHO pollution was further methodically evaluated, and the increased concentration of CO_2_ was closely monitored (Figures S11 and S12). As illustrated in Figure S13, the CO_2_ release content of the 2.0%Au/YT sample performs about 1179.7 ppm, which is 19.86‐times higher than that of the original ligand of H_4_TBAPy (59.4 ppm) and also much higher than the commercially available P25 (345.9 ppm). The main factors in this reaction were further confirmed by control variable tests conducted under various reaction circumstances, as illustrated in Figure S14. When absent the catalyst, no CO_2_ products were produced, demonstrating the acetaldehyde's structural stability. At the meantime, a minor amount of CO_2_ (105.3 ppm) was created in the absence of light, which is explained by the fact that this reaction was catalyzed by a tiny amount of ambient light. More significantly, the reaction is much more intense in an air environment (1179.7 ppm) than in an N_2_ atmosphere (689.5 ppm), suggesting that the reactive oxygen species produced by oxygen in this reaction are crucial for improving the efficiency of photocatalytic acetaldehyde pollutant purification. While, activity experiments with no catalyst completely showed no degradation, highlighting the crucial role of photocatalyst in driving the reaction. Furthermore, when the length of light exposure increased, an increasing trend of the electron paramagnetic resonance (EPR) intensity of 2.0%Au/YT emerged (Figure S15). This result further demonstrates that the superoxide (·O_2_^−^) radicals were crucial to the photocatalytic activities. Furthermore, the performance evaluation for CH_3_CHO removal under the same experimental circumstances was used to confirm the photocatalyst's structural integrity and recyclability after four reuse cycles (Figure S16).

The formation of toxic intermediates is a severe issue that still poses a significant obstacle to the widespread application of photocatalysts for air purification [36, 46]. Based on an in situ diffuse reflection infrared Fourier transform spectroscopy (DRIFTS) analysis, this work additionally monitored the chemical pathways associated in the photocatalytic advanced oxidation processes (AOPs) of CH_3_CHO in 2.0% Au/YT (Figure S17). It is evident that several distinctive signals associated with acetic acid (*ν*(–CH_3_) at 1370∼1470 cm^−1^, *ν*(O–C═O) at 1540∼1640 cm^−1^) were clearly visible when the lamp was turned on. However, the characteristic peaks of acetic acid first rise and then decrease as the reaction time increases, indicating that acetic acid is an intermediate product capable of further oxidation and mineralization to CO_2_ [47]. Nevertheless, during the oxidation degradation processes, the characteristic vibration band intensity of formaldehyde (*ν*(C═O) at 1710∼1810 cm^−1^) is gradually decreases, indicating that acetaldehyde can be photocatalyzed to form formaldehyde, which is subsequently further oxidized and transformed into the intermediate formic acid. More crucially, these intermediates created during the breakdown of acetaldehyde can be fully mineralized to carbon dioxide (*ν*(O═C═O) at 2280 ∼ 2390 cm^−1^) and water (*ν*(O–H) at 3680–3830 cm^−1^), since the unique peak of CO_2_ increases dramatically as the reaction proceeds. Lastly, based on the intermediate products found by in situ DRIFTS and HPLC analysis, the photocatalytic reaction routes for CH_3_CHO conversion were proposed in Figure S18.

### 3.5 DFT Calculations and Proposed Mechanism

To gain atomistic insights into the interfacial charge‑transfer behavior and reactant adsorption/ activation mechanisms of the Au/Y‑MOF system, density functional theory (DFT) calculations were performed, and a highly representative molecular cluster model was constructed [48, 49]. The model was built following three steps: (a) based on the experimental XRD refinement data, a supercell was first built, from which a surface fragment was extracted and the periodic boundary conditions were removed. This approach avoids the massive dangling bonds and artificial electronic distortion that would arise from cleaving periodic slab surfaces directly. Meanwhile, the fragment is rich in coordinatively unsaturated Y–O active sites. Boundary atoms were terminated to maintain the local electronic environment of the surface‐active sites; (b) an Au cluster (face‐centered cubic configuration) was placed on the substrate fragment surface for full structural optimization. The cluster size is consistent with the trend of the 2–3 nm of Au QDs observed by HR‑TEM, reasonably reflecting the cluster electronic structure under quantum‑size effects; (c) a vacuum layer was introduced along the Z direction to effectively eliminate unphysical electrostatic interactions between adjacent periodic images.

The crucial band reconstruction caused by Au cluster introduction is revealed by the overlap population, total and partial density of states (DOS) analysis from the standpoint of electronic structure (Figure 5a–c). The vertical dashed line indicates the HOMO level of the entire cluster. Pristine Y‐MOF demonstrates typical semiconducting properties, in which C 2p and ligand π orbitals mostly contribute to the valence band maximum, and carboxylate O 2p hybridization produces the conduction band minimum. Upon Au cluster anchoring, numerous new localized states derived from the Au 5d hybrid orbital emerge near the HOMO level, further crossing the HOMO level and penetrating into the band gap of the Y‑MOF, thereby conferring a metallic character at the interface. This significant enhancement of interfacial electronic density of states not only substantially lowers the contact resistance but also provides energetically degenerate “bridge” channels for the rapid transfer of photogenerated electrons from the Y‑MOF conduction band to the Au cluster, thus kinetically ensuring efficient charge separation. The calculated charge density difference clearly reveals the spatial charge redistribution at the interface. As visualized in Figure 5d–f, the results clearly show electron accumulation (represented by green regions) around the Au cluster and a corresponding electron depletion (blue regions) on the Y‐MOF surface, precisely confined to the Y atoms adjacent to the interface and the coordinating carboxylate O atoms. This provides direct theoretical evidence for the electron transfer direction from the Y‐MOF to the Au cluster upon their intimate contact, which is in perfect agreement with in situ XPS results, providing computational corroboration for the experimentally determined charge‑transfer dynamics.

**FIGURE 5:**
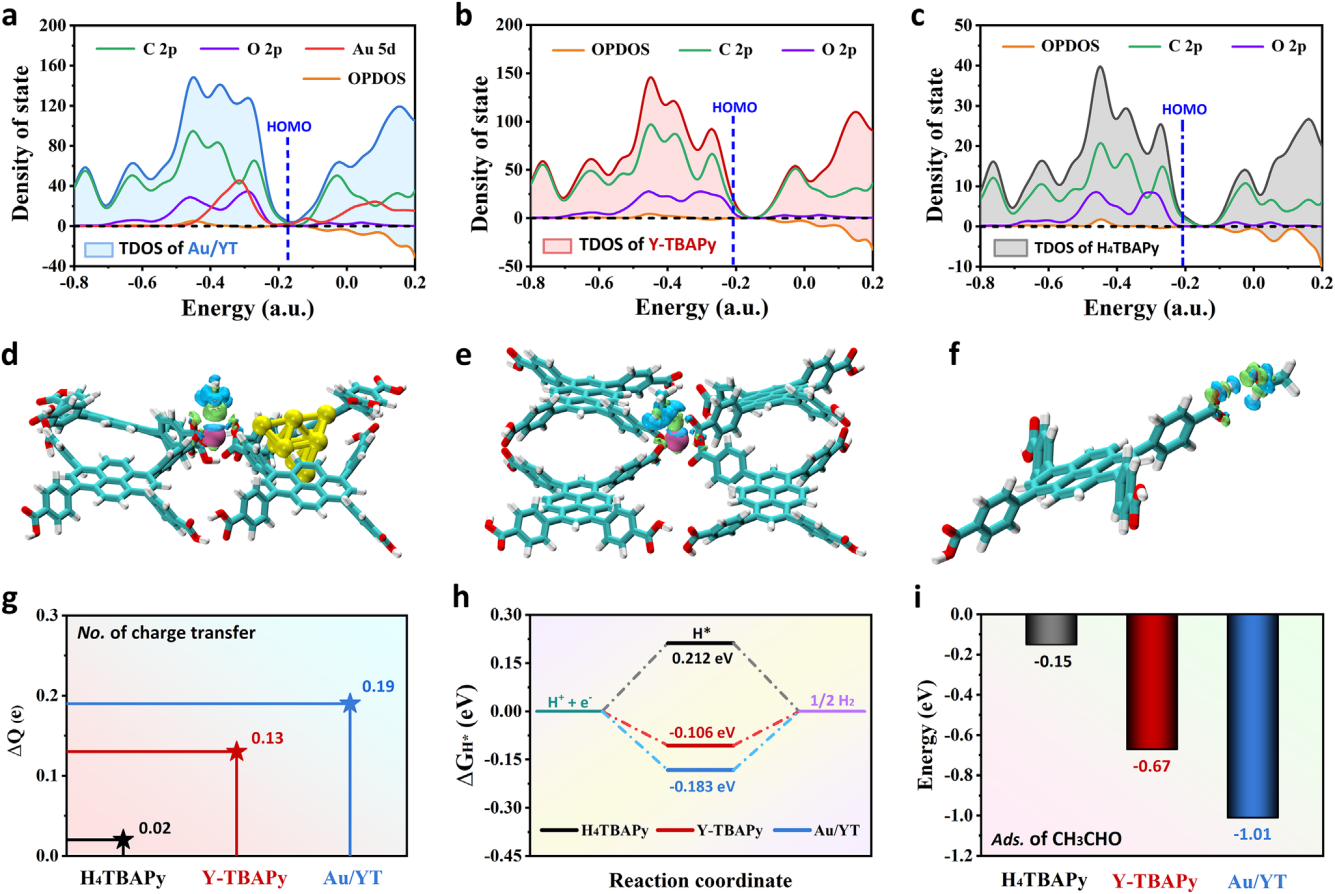
Overlap population, total and partial density of states (DOS) curves and calculated charge density difference plots (vertical dashed line represents the position of the HOMO level, isosurface value = 0.005 e·Å^−3^) of (a, d) Au/YT, (b, e) pristine Y‐TBAPy and (c, f) H_4_TBAPy, respectively; (g) Calculated number of charge transfer (ΔQ); (h) Gibbs free energy (ΔG) for hydrogen evolution reaction (HER) pathway; and (i) Quantitative adsorption energy of CH_3_CHO molecule.

In addition, the quantized charge transfer behavior between the different catalytic systems was further explored (Figure 5g). Charge analysis reveals that the charge transfer amount into the H_4_TBAPy substrate is only 0.02 q, while pristine Y‐MOF achieves 0.13 q. Quantitative Bader charge analysis further indicates that after Au nanocluster introduction, the Au/YT system exhibits a further increased charge transfer of 0.19 q, while the nearby Y–O coordination units lose an equivalent number of electrons, indicating superior interfacial charge exchange capability. This result confirms that the introduction of Au nanoclusters effectively modulates the electronic structure of the Y‐MOF through metal‑support interaction (MSI), increasing the electron density around the metal active sites and optimizing the charge distribution within the system.

To accurately evaluate the intrinsic hydrogen evolution reaction (HER) catalytic activity of the three systems, namely Au QDs anchored Y‐MOF (Au/YT), pristine Y‐TBAPy, and H_4_TBAPy, the Gibbs free energy of hydrogen adsorption (ΔG) on each surface was systematically calculated (Figure 5h). This parameter serves as a key descriptor for assessing the adsorption‑activation capability of hydrogen intermediates and hence the HER performance [50, 51]. Generally, a ΔG_H*_ value close to 0 eV is considered favorable for HER, and systems with ΔG_H*_ within approximately 0.2–0.3 eV are often regarded as promising candidates in preliminary DFT‐based HER screening [52, 53]. The H_4_TBAPy exhibited a ΔG_H*_ value of +0.212 eV, indicating weak hydrogen adsorption and insufficient activation of hydrogen intermediates, which limits the HER process. Pristine Y‐TBAPy displays a ΔG_H*_ value of –0.106 eV, demonstrating substantially enhanced hydrogen adsorption and effective capture/activation of hydrogen intermediates. The Au/YT system showed a further lowered ΔG_H*_ value of –0.183 eV, which remains within the near‐thermoneutral screening range and suggests favorable hydrogen adsorption among the three systems. Detailed analysis reveals that a pronounced MSI between Au nanoclusters and the Y‐MOF support effectively modulates the electron density and the electronic structure of the surface‐active sites, thereby enhancing hydrogen intermediate adsorption and activation. It should be noted that although the hydrogen adsorption strength on Au/YT is slightly higher than that on pristine Y‐TBAPy, the absolute value of ΔG_H*_ is still below 0.3 eV, indicating that H* adsorption is not expected to cause severe thermodynamic trapping. Collectively, although ΔG_H*_ alone should not be taken as complete kinetic proof of HER activity, the Au/YT system can provide a favorable thermodynamic environment for adsorption and activation of hydrogen intermediates.

DFT simulations were further used to predict CH_3_CHO adsorption on Au/YT, pristine Y‐MOF, and H_4_TBAPy catalyst systems in order to explore the impact during subsequent photocatalytic processes. The CH_3_CHO molecule was positioned to adsorb close to the Au sites in Au/YT. Quantitative adsorption energy analysis further demonstrates that anchored Au sites significantly enhance the adsorption stability of CH_3_CHO (Figure 5i). The calculated adsorption energy (E) for CH_3_CHO on H_4_TBAPy, pristine Y‐MOF, and Au/YT are −0.15, −0.67, and −1.01 eV, respectively. The negative values confirm spontaneous formation of stable adsorption configurations on all three systems, and the interfacial interaction between Y‐MOF and CH_3_CHO is substantially strengthened after Au QDs decoration, which may provide a thermodynamic basis for subsequent catalytic conversion of CH_3_CHO. As a result, CH_3_CHO undergoes more pronounced charge rearrangement and electron transfer during adsorption, facilitating the efficient generation of active intermediates. It should also be noted that adsorption energy alone cannot fully determine the overall reaction kinetics, because relatively strong adsorption may lead to intermediate retention if the subsequent transformation or desorption is unfavorable. Meanwhile, the optimized charge transfer environment stabilizes adsorbed intermediates, which is beneficial for CH_3_CHO activation but should be interpreted as thermodynamic evidence rather than direct proof of the complete conversion pathway. In summary, the electronic modulation induced by Au QDs concurrently enhances both CH_3_CHO conversion (via improved charge transfer and adsorption stability) and HER (via favorable hydrogen adsorption free energy) on the Y‐MOF catalyst, demonstrating a synergistic effect arising from the MSI. This work offers a theoretical and experimental basis for developing bifunctional MOF‑based photocatalysts for efficient environmental pollutant oxidation and hydrogen production.

Based on the observational characterization results and computational studies of the Au/YT photocatalyst, a synergistic photocatalytic redox reaction mechanism for the transformation of water into H_2_ along with toxic CH_3_CHO degradation is proposed. Typically, the pyrene moiety in the TBAPy^4−^ ligand has a planar structure with a large number of π electrons, forming a π‐conjugated system that strengthens photo‐induced electron transmission [23, 25]. The parallel arrangement of these pyrene units further promotes orbital overlap and strong electrical coupling. The integration of yttrium ions with TBAPy^4−^ ligands enhances the generation of photoinduced electrons, promoting effective separation of charges at the Y‐MOF interface. The photogenerated electrons are swiftly transferred from the Y–O clusters through the carboxylate linkers to the Au QDs, which function as efficient electron reservoirs, thereby prolonging the photogenerated carrier (dissociated from excitons) lifetime and suppressing recombination. The accumulated electrons at the Au QD sites subsequently reduce protons (H^+^) to H_2_, while the photogenerated holes are consumed by the oxidation of toxic CH_3_CHO, ultimately yielding non‐toxic CO_2_ molecules; the final proposed mechanism for green hydrogen evolution coupled with toxic acetaldehyde purification by the Au/YT photocatalyst is well illustrated in Figure 6 and Figure S19. Thus, the elaborated Au quantum dots‐decorated Y‐MOFs bifunctional photocatalyst in this study has been proved to be a good synergistic photocatalysis candidate for energy conversion and environmental remediation‐related applications.

**FIGURE 6:**
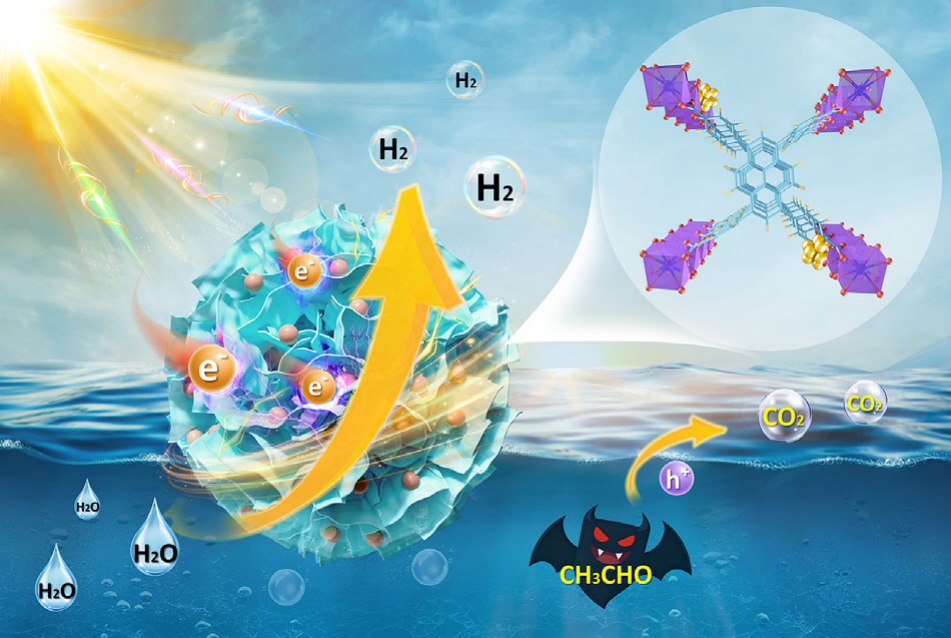
The proposed mechanism by Au QDs‐decorated Y‐MOF for redox synergistic photocatalysis applications of green hydrogen evolution and toxic acetaldehyde purification.

## 4 Conclusions

In summary, we successfully fabricated a novel dual‐functional Au/YT photocatalyst via a straightforward one‐pot solvothermal approach, wherein Au quantum dots are securely anchored onto an yttrium‐based pyrene‐derived MOFs. An integrated experimental and theoretical investigation unequivocally demonstrates that the Au QDs critically orchestrate the electronic properties of the Au/YT photocatalyst. The robust interfacial electronic coupling establishes a directional charge‐transfer highway, wherein Au QDs can act as efficient electron reservoirs to curtail charge recombination and concurrently provide optimized active sites for surface redox photocatalysis. Consequently, the optimal 2.0%Au/YT manifests an outstanding enhancement in H_2_ production alongside exceptional efficiency in CH_3_CHO mineralization. This work provides a compelling paradigm demonstrating that strategic Au QDs functionalization can effectively transform a pristine MOFs into a high‐performance bifunctional platform for coupled solar‐to‐fuel conversion and environmental remediation, thereby paving the way for the rational design of advanced MOFs‐based photocatalysts.

## Author Contributions

**Hui Yang**: Writing – original draft, Conceptualization, Funding acquisition, Investigation. **Dong Xu**, **Xue Lu** and **Yinbao Wu**: Investigation, Data curation, Formal analysis. **Feng Tang**, **Linfu Xie** and **Saisai Yuan**: Software, Methodology. **Xiaozhi Wang**, **Qitao Zhang** and **Bin Xu**: Writing – review & editing, Conceptualization, Supervision, Funding acquisition, Validation. Project administration. All authors have given approval to the final version of this paper.

## Conflicts of Interest

The authors declare no conflicts of interest.

## Supporting information




**Supporting File**: advs78695‐sup‐0001‐SuppMat.docx.

## Data Availability

The data that support the findings of this study are available from the corresponding author upon reasonable request.
