# Supplementary material for: Au Quantum Dots Anchored Y‐MOF Enabling Directional Interfacial Charge Transfer for Synergistic Redox Photocatalysis
Source: Adv Sci (Weinh). 2026 Nov 5:e78695. Online ahead of print. doi: 10.1002/advs.78695 (PMC13735439; doi:10.1002/advs.78695)
Supplement: Supplementary file 1 [file ADVS-9999-e78695-s001.docx]

**Supporting Information**

**Au Quantum Dots Anchored Y-MOF Enabling Directional Interfacial Charge Transfer for Synergistic Redox Photocatalysis**

**Hui Yang ^a, b^, Dong Xu ^a^, Xue Lu ^a^, Yinbao Wu ^a^, Feng Tang ^c^,** **Linfu Xie ^c^, Saisai Yuan ^d^, Xiaozhi Wang ^a^, Qitao Zhang ^c,^** ***, Bin Xu ^b,^** *

**^a^** School of Environmental Science and Engineering, Yangzhou University, Yangzhou 225127, China

**^b^** School of Chemistry and Materials, Yangzhou University, Yangzhou 225002, China

**^c^** International Collaborative Laboratory of 2D Materials for Optoelectronics Science and Technology of Ministry of Education, Institute of Microscale Optoelectronics, Shenzhen University, Shenzhen 518000, China

**^d^** School of Environmental and Chemical Engineering, Jiangsu University of Science and Technology, Zhenjiang 212000, China

* **Corresponding authors.**

**E-mail addresses: qitao-zhang@szu.edu.cn (Q. Zhang); twt@yzu.edu.cn (B. Xu)**

**S1. Experimental Section**

**S1.1 Chemicals**

All chemicals and reagents were of analytical grade and obtained from Shanghai Aladdin Biochemical Technology Co., Ltd, China. Mainly including 1, 3, 6, 8-tetra(4-carboxyl phenyl) pyrene (H_4_TBAPy, ≥97%), yttrium nitrate (Y(NO_3_)_3_), gold(III) chloride hydrate (HAuCl_4·_XH_2_O), iodine (I_2_), 1, 4-dioxane, N, N-dimethylformamide (DMF), acetaldehyde (CH_3_CHO, ≥99.5%), sodium sulfate (Na_2_SO_4_), Nafion solution, 1, 4-benzoquinone (BQ), methanol (MeOH) and silver nitrate (AgNO_3_) were used without any further purification. In addition, deionized water self-made in the laboratory was used throughout the experiments.

**S1.2 Synthesis method of pristine Y-TBAPy photocatalyst**

At first, 1, 3, 6, 8-tetra(4-carboxyl phenyl) pyrene (H_4_TBAPy, 136.53 mg, 0.2 mmol) and yttrium nitrate (Y(NO_3_)_3_, 114.87 mg, 0.3 mmol) were co-dissolved into a mixed solvent of 1, 4-dioxane/DMF/ H_2_O (20.0 mL, volume ratio of 1:3:1) and magnetically stirred at 25℃ for 3 hours. Afterwards, the stirred solution was then transferred to a 50 mL PTFE-lined stainless-steel reactor and heated at 120℃ for 72 hours. Upon completion of the solvothermal reaction, the product was washed with a DMF/H_2_O mixed solution (volume ratio of 1:1), and centrifuged at 10000 rpm for 15 minutes every time. Afterwards, this wash action subjected at least three times, and then dried at 80℃ for 12 hours to yield the yellow powder denoted as Y-TBAPy (YT).

In addition, before the activity test, the photocatalyst was irradiated under a UV lamp for 12 hours to remove organic adsorbates on the photocatalyst surface.

**S1.3 Characterization instruments of materials**

The crystal structure of catalysts was determined using an X-ray diffractometer (XRD, Rigaku Ultima IV) in a 2θ range from 3° to 80°. The metal ion contents were determined using an inductively coupled plasma optical emission spectrometry (ICP-OES, Agilent Technologies). Fourier transform infrared spectra (FT-IR) were measured with a Nicolet iS20 spectrometer (Thermo Scientific) using the KBr tableting method. Morphology information and elemental mapping images of the products were observed by high resolution transmission electron microscopy (HR-TEM, FEI, Tecnai G2F30S-TWIN, 300 KV) and scanning electron microscopy (SEM, JEOL, JSM-6701F) technology. The surface chemical composition and valence states of the photocatalysts were determined by X-ray photoelectron spectroscopy (XPS) on a K-Alpha spectrometer (Thermo Scientific). *In-situ* XPS measurement was conducted under a 300 W Xe lamp used as the light source (λ≥420 nm, light intensity of 100 mW·cm^-2^), and the test temperature was maintained at 298 K. The light absorption property of the samples was analyzed using ultraviolet-visible spectrophotometry (UV-Vis, Persee TU-1901). Time-resolved photoluminescence spectra (TRPL) of samples were recorded on an Edinburg Instruments FLS920 spectrofluorometer at room temperature with a 300 nm nanosecond laser. The photoluminescence (PL) spectra were obtained using an FLS1000 spectrophotometer (Edinburgh). The temperature-dependent photoluminescence (TD-PL) spectra were tested on an Edinburg Instruments FLS920 spectrofluorometer from 10 to 300 K under 325 nm laser excitation.

**S1.4 Photoreduction experiment of H_2_ evolution**

Photocatalytic hydrogen evolution reaction (HER) of water splitting was completed in a glass vessel connected to an automatic online trace gas circulation system (Beijing Perfectlight, Labsolar-6A). For each experiment, 20 mg of photocatalyst was dispersed into 40 mL H_2_O solution containing 4 mL triethanolamine (TEOA) as sacrificial agent. The reaction temperature should be kept below 15℃ and the condensate water circulation system should be continuously adopted. A 300 W Xe lamp (Beijing Perfectlight, PLS-SXE300D) adding a 420-nm cutoff filter was used as the light source. The generated concentration of H_2_ was tested by real-time gas chromatography (FULI, GC9790 Plus, TCD detector).

**S1.5 Photocatalytic experiment of CH_3_CHO removal**

Photooxidation performance of photocatalysts was evaluated by measuring the decomposition of acetaldehyde gas (CH_3_CHO, Wako Chemical Reagent Co. Ltd.). Initially, a 3 cm diameter glass dish was placed in a 500 mL volume Tedlar bag with 20 mg of photocatalyst powder applied to the bottom of the dish. Next, a concentration of 600 ppm gaseous CH_3_CHO and synthetic air were introduced into the Tedlar bag. To achieve an adsorption-desorption equilibrium, this bag was then placed in a dark environment for 2 hours. Then, a Xe lamp (PXE-500, USHIO Inc.) with a 420 nm cut-off filter was used as light source. Lastly, an online gas chromatograph (Agilent Technology 3000 A Micro-GC, FID detector) was used to monitor the amount of CO_2_ produced in real time. Moreover, after one cycling test, the 2.0%Au/YT photocatalyst was irradiated under a UV lamp for 8 hours to remove organic adsorbates or impurities on the photocatalyst surface for further cycle use.

**S1.6 Transient photo****current response measurements**

A suspension was prepared by ultrasonication of 10 mg photocatalyst powder in 10 mL acetone within an amber glass vial for 30-minutes. Subsequently, 5 mg iodine (I_2_) was added to the mixture, followed by an additional 30-minutes ultrasonication. The resultant suspension was deposited onto indium tin oxide (ITO)-coated glass substrates (an active area of 1×1 cm^2^) using a constant-voltage electrochemical deposition system. The working electrode was irradiated by a Xenon lamp source (Beijing Perfectlight Co. Ltd, PLS-SXE300D) with a 420-nm filter cutoff. Next, a three-electrode system was consisting of Ag (calibrated against Ag/AgCl) as reference electrode, Pt electrode as counter electrode and photocatalyst loaded ITO conductive glass as working electrode. Finaly, photocurrent test was impregnated in saturated Na_2_SO_4_ solution (0.1 mol/L, pH=6.8) with an electrochemical analyzer (CHI 760D, Shanghai, Chenhua). The applied voltage controlled at 0.2 V (determined by “open circuit potential” operation). Meanwhile, a shutter was used to control the light switch every 20 seconds to test the photocurrent response data.

**S1.7 Electrochemical impedance spectroscopy (EIS) tests**

A photocatalyst ink was formulated by dispersing 5 mg photocatalyst powder in 250 μL ethanol with 5 μL Nafion solution under 20-minutes ultrasonication. Concurrently, a glassy carbon electrode (GCE) was polished to a mirror finish using 0.05 μm alumina slurry, followed by thorough rinsing with deionized water. The photocatalyst ink was drop-cast onto the pretreated GCE surface and dried under ambient conditions. This coating procedure was repeated for 2–3 cycles to achieve uniform photocatalyst loading. The working electrode was put in the saturated Na_2_SO_4_ electrolyte (0.1 mol/L, pH=6.8) and irradiated by a Xenon lamp source (Beijing Perfectlight Co. Ltd, PLS-SXE300D) with a 420 nm filter cutoff. Lastly, EIS tests were conducted on the CHI 760D electrochemical analyzer, at open-circuit potential of 0.2 V vs. Ag/AgCl, with AC perturbation amplitude of 10 mV, and over the frequency range 0.1 Hz to100 kHz.

**S2. Computational details**

All quantum chemical calculations were conducted employing the Gaussian 16 software package. For the pristine Y-MOF system (without Au cluster modification), the geometries of MOF-adsorbed acetaldehyde complexes were fully optimized and subsequently analyzed for intermolecular interactions using density functional theory (DFT) at the B3LYP/6-31G* level. In contrast, for the Au-anchored Y-MOF system, a mixed basis set approach was meticulously implemented: the Au cluster was described with the relativistic ally-corrected Lanl2DZ effective core potential (ECP) basis set, while all other atoms (carbon, oxygen and hydrogen) were treated with the standard 6-31G* basis set. The binding interactions were thoroughly analyzed employing the Integrated Gradient Molecular (IGM) approach, which enables clear visualization of non-covalent interactions. The resultant isosurface maps were generated and visualized in VMD by utilizing cube files exported from Multiwfn 3.8, in which the comprehensive and accurate representation of the electronic structure information was ensured.

Additionally, geometry optimizations were performed for both the pristine Y-MOF and Au/Y-MOF structures to evaluate the full reaction pathway of the H_2_ production. The thermodynamic energy corrections to the Gibbs energy were evaluated at 298.15 K and 1 atm.

The adsorption energy (E_Ads_) was calculated by using the Eq. S1:

E_Ads_ = E_Total_ – E_Substrate_ – E_Adsorbate_	(S1)

Where E_Total_ is the energy of adsorption structure, E_Subtrate_ is the energy of the substrate, and E_Adsorbate_ is the energy of the adsorbate (CH_3_CHO).

The Gibbs free energy change (ΔG) of each chemical reaction was calculated by Eq. S2:

ΔG = ΔE + ΔZPE – TΔS	(S2)

Where E is the calculated total energy, ZPE is the zero-point energy, T is the temperature, and S is the entropy.

**S3.** **Electron/hole balance calculations**

Since the complete oxidation of CH_3_CHO releases 10e⁻ per molecule (CH_3_CHO+3H_2_O → 2CO_2_+10H⁺+10e⁻), while H_2_ evolution consumes 2e⁻ per molecule (2H⁺+2e⁻→ H_2_), so the theoretical H_2_/CH_3_CHO stoichiometry is 5:1 for full mineralization.

The electron balance between H_2_ evolution and CH_3_CHO oxidation was evaluated under identical conditions (Ar-purged sealed reactor, 15°C, all CO_2_ and carbonate-C values were blank-subtracted). Typically, 20.0 mg catalyst dispersed in 40.0 mL of H_2_O solution containing 0.4 mL of CH_3_CHO produced 16.44 µmol of H_2_ (821.8 µmol g⁻¹) within 2 h, consuming 32.87 µmol of electrons on the reduction side (2e⁻ per H_2_). On the oxidation side, the quantified products comprised 335.3 ppm of CO_2_ (6.38 µmol, i.e. 31.91 µmol e⁻; converted using a headspace volume of 0.45 L at 1 atm and 15℃) and 1.98 µmol dissolved carbonate/bicarbonate (i.e. 0.0496×10^-3^ mol/L, i.e. 9.92 µmol e⁻), whose presence was confirmed by CaCl_2_/HCl titration.

Defining the electron balance ratio as R=2*n*(H_2_)/[5*n*(CO_2_+ carbonate-C)], with 5e⁻ counted per carbon for complete mineralization, the quantified oxidation products account for 41.83 µmol e⁻, exceeding the 32.87 µmol e⁻ consumed by H_2_ evolution by 8.96 µmol e⁻ (27.3% relative to the reduction side); **hence R = 0.786 (78.6%), i.e. the electrons consumed by H_2_ evolution amount to 78.6% of those released by the quantified oxidation products.** The deviation of R from unity may attributed to the reduction side reactions of trace dissolved oxygen, which consumed additional photogenerated electrons. Accordingly, R<1 demonstrates that the electrons consumed by H_2_ evolution are already fully covered by the quantified CH_3_CHO oxidation products, with the excess oxidation being attributable to intermediate formation; this confirms that H_2_ evolution and CH_3_CHO oxidation are electronically coupled with a closed electron/hole balance.

**S4. Supplementary Figures**

**Figure S1.** Coordination structure of **a** H_4_TBAPy ligand, **b** pristine Y-TBAPy and **c** Au/YT.

**Figure S2.** HAADF and elements distribution mapping images of pristine Y-TBAPy.

**Figure S3.** High-resolution XPS spectra of **a** C 1s, **b** O 1s, and **c** Y 3d on Y-TBAPy.

**Figure S4.** Tauc plot curves for the band gap calculation on YT-based photocatalysts with different Au QDs loading ratios.

**Figure S5.** Mott-Schottky (M-S) plot and flat-band potential of 2.0%Au/YT photocatalyst.

**Figure S6.** Steady PL spectra of 2.0%Au/YT and pristine Y-TBAPy samples.

**Figure S7.** Comparation of H_2_ production rate before and after adding Pt as co-catalyst.

**Figure S8.** Recycling tests of H_2_ evolution on the optimal 2.0%Au/YT photocatalyst.

**Figure S9.** XRD pattern of 2.0%Au/YT photocatalyst before and after photocatalytic reactions.

**Figure S10.** XPS Au 4f spectra of 2.0%Au/YT before and after photocatalytic reactions.

**Figure S11.** Photocatalytic CH_3_CHO consumption concentration-time curves over YT-based photocatalysts with different Au QDs loading ratios.

**Figure S12.** Photocatalytic CO_2_ generation concentration-time curves performance of over YT-based photocatalysts with different Au QDs loading ratios.

**Figure S13.** Comparation of CO_2_ generated amount from CH_3_CHO conversion.

**Figure S14.** Control experiments of different reaction conditions on 2.0%Au/YT photocatalyst.

**Figure S15.** EPR signals of superoxide radicals (·O_2_^-^) on 2.0%Au/YT and pristine Y-TBAPy samples.

**Figure S16.** Cycling tests of CH_3_CHO degradation to generate CO_2_ on 2.0%Au/YT sample.

**Figure S17.** *In-situ* DRIFTS analysis for photocatalytic purification process of CH_3_CHO pollution on the 2.0%Au/YT photocatalyst.

**Figure S18.** Proposed conversion pathways of CH_3_CHO removal on 2.0%Au/YT photocatalyst.

**Figure S19.** Proposed mechanism of synergistic redox reactions on the Au/YT photocatalyst.

**Table S1.** ICP-OES measurement to determine the actual Au loadings.

| **Samples** | **Au/wt%** |
| --- | --- |
| **0.5%Au/YT** | **0.34** |
| **1.0%Au/YT** | **0.71** |
| **2.0%Au/YT** | **1.43** |
| **3.0%Au/YT** | **2.11** |

**Table S2.** The fitted parameters obtained from EIS Nyquist plots.

| **Samples** | **R_s_ (Ω)** | **R_ct_ (Ω)** | **CPE-P (n)** | **(1−n)·90°** | **RMS (Ω)** |
| --- | --- | --- | --- | --- | --- |
| **2.0%Au/YT** | **≈0** | **69.1** | **0.84** | **14** | **2.3** |
| **Y-TBAPy** | **≈0** | **115.8** | **0.85** | **13** | **2.2** |
| **H_4_TBAPy** | **≈0** | **160.0** | **0.93** | **6** | **4.2** |

**Table S****3.** Comparison of H_2_ evolution performance of reported Au/MOF-based photocatalysts.

| **Catalyst** | **Light source** | **Reaction solution** | **H_2_ evolution (μmol·g⁻¹·h⁻¹)** | **Ref.** |
| --- | --- | --- | --- | --- |
| Au@NH_2_-UiO-66/CdS | Xe lamp  (λ>420 nm) | Acetonitrile/lactic acid/H_2_O | 664.9 | [S1] |
| UCNPs-Pt@MOF/Au | simulated solar | Acetonitrile/ TEOA/H_2_O | 280 | [S2] |
| Au/UiO-66-NH₂ | Xe arc lamp | Methanol solution | 387 | [S3] |
| Au/PFC-9 | Xe lamp  (λ>400 nm) | TEOA/H_2_O | 130 | [S4] |
| 2.0%Au/YT | Xe lamp  (λ>420 nm) | TEOA/H_2_O | 508.5 | **This work** |

**Table S4.** Comparison of pollution degradation activity with that of other reported photocatalysts.

| **Catalyst** | **Light source** | **Target** | **Efficiency** | **Ref.** |
| --- | --- | --- | --- | --- |
| Au–TiO_2_@NH_2_-UiO-66 | Xenon lamp | Ethyl acetate | 94.6% | [S5] |
| Au–WO_3_/TiO_2_ | LED light (365 nm) | Toluene | 95.4% | [S6] |
| TM-ZrO_2_@NH_3_-UiO-66 | 300 W Xe (350–780) | Formaldehyde | 80% | [S7] |
| AUiO-66(Zr/Ti) | Xe lamp  (λ>400 nm) | Toluene | 83% | [S8] |
| 2.0%Au/YT | Xe lamp  (λ>420 nm) | Acetaldehyde | 98.3% | **This work** |

S1. Z. Li, J. Zi, X. Luan, Y. Zhong, M. Qu, Y. Wang, Z. Lian, Localized surface plasmon resonance promotes metal-organic framework-based photocatalytic hydrogen evolution, ***Adv. Funct. Mater.*** 33 (2023) 2303069, <https://doi.org/10.1002/adfm.202303069>.
S2. D. Li, S.-H. Yu, H.-L. Jiang, From UV to near-infrared light-responsive metal-organic framework composites: plasmon and upconversion enhanced photocatalysis, ***Adv. Mater.*** 30 (2018) 1707377, <https://doi.org/10.1002/adma.201707377>.
S3. S. Subudhi, K. Parida, Zr-based MOF: an enhanced photocatalytic application towards H₂ evolution by consequence of functional group and LSPR effect, ***Mater. Today Proc.*** 35 (2021) 198-202, <https://doi.org/10.1016/j.matpr.2020.04.252>.
S4. Q.-Q. Huang, Z.-B. Fang, K. Pang, W.-K. Qin, T.-F. Liu, R. Cao, The impact of secondary building units in metal-organic frameworks on plasmonic gold-sensitized photocatalysis, ***Adv. Funct. Mater.*** 32 (2022) 2205147, <https://doi.org/10.1002/adfm.202205147>.
S5. H. Liu, X. Chang, X. Liu, G. Li, W. Zhang, T. An, Boosting the photocatalytic degradation of ethyl acetate by a Z-scheme Au-TiO_2_@NH_2_-UiO-66 heterojunction with ultrafine Au as an electron mediator, ***Environ. Sci.: Nano*** 8 (2021) 2542-2553, [https://doi.org/10.1039/ d1en00225b](https://doi.org/10.1039/%20d1en00225b).
S6. X. Wang, H. Pan, M. Sun, Y. Zhang, Au single atom-anchored WO_3_/TiO_2_ nanotubes for the photocatalytic degradation of volatile organic compounds, ***J. Mater. Chem. A*** 10 (2022) 6078-6085, <https://doi.org/10.1039/d1ta08143h>.
S7. J. He, X. Yu, X. Luan, H. Li, S.J. Shah, W. Su, Z. Jia, J. Chen, L. Zhou, J. Deng, Z. Zhang, Z. Huang, Z. Zhao, Facile construction of tourmaline@MOFs composites via micron-scale electrical polarization to enhance photodegradation of gaseous formaldehyde, ***Chem. Eng. J.*** 479 (2024) 147828, <https://doi.org/10.1016/j.cej.2023.147828>.
S8. L.-Y. Lin, C. Liu, V.D. Dang, H.-T. Fu, Atomically dispersed Ti-O clusters anchored on NH_2_-UiO-66(Zr) as efficient and deactivation-resistant photocatalyst for abatement of gaseous toluene under visible light, ***J. Colloid Interface Sci.*** 635 (2023) 323-335, [https://doi.org/10.1016/j. jcis.2022.12.147](https://doi.org/10.1016/j.%20jcis.2022.12.147).
